# Synthesis and molecular docking analysis of MBH adducts' derived amides as potential *β-lactamase* inhibitors

**DOI:** 10.6026/973206300200449

**Published:** 2024-05-31

**Authors:** Hamid Ullah, Sadia Majid, Asma Abro, Taj Ur Rahman, Abdul Majeed Khan, Mehboob Ahmed, Muhammad Asif, Asma Yousafzai, Riffat Ullh, Peter Natesan Pushparaj, Mahmood Rasool

**Affiliations:** 1 Department of Chemistry, Balochistan University of Information Technology, Engineering and Management Sciences, Takatu Campus, Quetta 87300, Pakistan; 2 Department of Chemistry, Mohi-Ud-Din Islamic University Nerian Sharif, AJ & K, Pakistan; 3 Department of Biotechnology, BUITEMS, Takatu Campus, Quetta, Pakistan; 4 General Studies Department, Jubail Industrial College, Jubail Industrial City 31961, Saudi Arabia; 5 Department of Biotechnology & ORIC, BUITEMS, Takatu Campus, Quetta, Pakistan; 6 H. E. J. Research institute of chemistry, ICCBS, University of Karachi, Pakistan; 7 Center of Excellence in Genomic Medicine Research, Faculty of Applied Medical Sciences, King Abdulaziz University, Jeddah 21589, Saudi Arabia

**Keywords:** Synthesis, MBH adducts, antibacterial amides, ADME properties, *in silico* studies, *β-lactamase* inhibitors

## Abstract

Humans suffer from various diseases that require more specific drugs to target them. Among the different potent agents, *β-lactamase*s serve as good antibacterial agents; however, *β-lactamase*s are resistant to such antibiotics. The present study was designed to prepare efficient *β-lactamase* inhibitor amides (12-15) from inexpensive, easily accessible, and bioactive precursors; Morita Baylis Hillman (MBH) adducts (5-8). The adducts (5-8) were primarily prepared by treating their respective aldehydes with the corresponding acrylate in the presence of an organic Lewis base at ambient temperature. The compounds were characterized using mass spectrometry, FTIR and NMR spectroscopy. Furthermore, *in silico* studies (using AutoDock Tools and AutoDock Vina programs) on the adduct and corresponding amide product revealed that all MBH adducts (5-8) and their product amides (12-15) are significant inhibitors of *β-lactamase*. Additionally, among the MBH adducts, adduct 7 showed the highest binding affinity with *β-lactamase*, whereas amide 15 was identified as a highly potent antibacterial based on its docking score (-8.6). In addition, the absorption, distribution, metabolism, and excretion (ADME) test of the synthesized compounds demonstrated that all compounds showed drug-likeness properties.

## Background:

**Figure 1:**
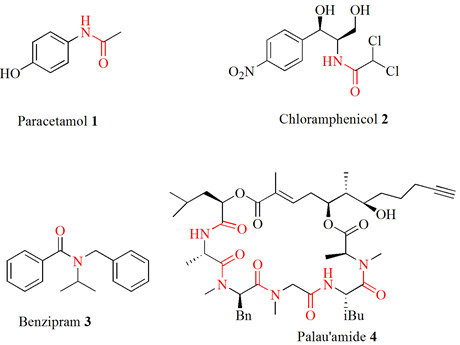
Structural formulae of compounds 3-4.

Carboxylic acid-derived organic substances bearing the general formula "RC (=O) N R1R2" are termed as amides. The amide bond is mainly found in organic compounds and biomolecules owing to its high stability because of the resonance and its stability under high-temperature reaction conditions. These building blocks are highly important from a chemical perspective because of their hydrogen-bond accepting and donating properties [1, 2]. Their application in chemistry and biology is enormous because they can be used in the synthesis of a variety of other important compounds, such as the synthesis of amide-linked imidazopyridine derivatives exhibiting anticancer activity, and capsaicin (a natural amide and an active component from chili pepper) and its derivatives exhibiting antioxidant and antibacterial activities have been reported in the literature [3, 4]. Carboxamides are necessary for maintaining life, because proteins and peptides are polyamides. Amide structures containing various natural and synthetic organic molecules are used in medicine and agrochemicals. For example, L-dopa-derived amides are more effective against Parkinson's disease than their precursor L-dopa [5]. Some biologically active amide-containing compounds are shown in Figure 1.

**Figure 2:**
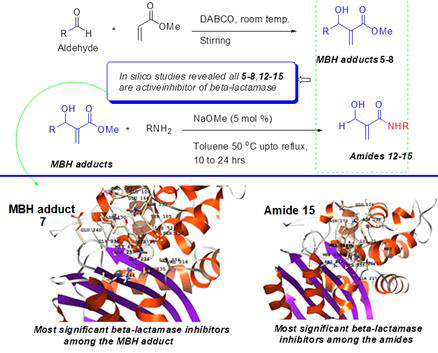
An infographical representation of the current study indicating the synthetic scheme and the most efficient *β-lactamase* inhibitors.

The literature reveals that more than 25% of known drugs have carboxamide functionality [6]. Owing to their great importance, various efforts have been made to achieve these targets with diverse functionalities and using different strategies, including the Passerini, Ritter, Schmidt, and Ugi reactions. The literature also reports the use of various substrates in the synthesis of amides, including aryl halides, oximes, nitriles, carboxylic acids, and esters. Various methods have been reported to convert ester precursors into amides that include DBU catalysed aminolysis [7, 8]. Another method involves ester conversion into respective acid halides and subsequent aminolysis to respective amides, catalyzed by an alkoxide base [9]. Additionally, esters can be converted into amides via ruthenium-catalyzed aminolysis [10]. Esters are the most commonly used precursors for amide preparation. However, no example has yet been reported for the utilization of Morita Baylis Hillman (MBH) adducts as ester precursors to achieve amides. Because MBH adducts can easily be obtained under mild conditions and display great synthetic applicability [11, 12], this study aimed to check their synthetic application in the preparation of the most interesting class of organic compounds. Amide derivatives are also known to be chemically reactive to penicillins, which are used as antibiotics. *β -lactamases* specifically recognize β-lactam antibiotics and act as defense enzymes for many drug-resistant bacteria. The design of *β-lactamase* inhibitors has been adopted as a common strategy against enzymatic action to address drug resistance [13]. Therefore, it is of interest to describe the synthesis of MBH adducts, their transformation into amides, and the subsequent molecular modelling study of the synthesized compounds with *β-lactamase* enzyme and discusses the binding mechanism. In a summarized way, the present study is presented in Figure 2.

## Experimental:

## General procedure:

The column chromatography method was adopted for the purification/isolation of natural substances. The solvents and chemicals were of synthetic grade and obtained from Sigma-Aldrich. UV lamp and TLC card coated with silica gel 60 F254 on aluminum sheets (Merck) were used for the pre-identification of the synthesized compounds. Additionally, an analytical balance, oven, oil bath, constant temperature hot plate and stirrer, Gallenkamp melting point apparatus, FTIR spectrophotometer (Shimadzu), and NMR spectrophotometer were used.

## Procedures for preparation of MBH adduct 5-8:

In each reaction, the respective aldehydes (1 equivalent) were placed in a 50-100 mL round bottom flask. It was added 5 equivalents of methyl acrylate and 0.65 equivalents of DABCO and then the reaction mixture was sonicated for several hours (Time varied, depended mostly on aldehydes) at room temperature. The reaction progress was monitored using a TLC card, and possible active spots were observed under a UV lamp. When the reactant spots on the TLC card disappeared and a relatively new, more polar spot appeared on the card, this point was considered the reaction completion point. Thus, sonication was stopped at this point, and the reaction was subjected to a workup. The excess acrylate was removed under reduced pressure, resulting in gummy crude. A saturated solution of brine and ethyl acetate (10-15 mL) was then added. The two resulting layers (organic and inorganic) were separated using a separating funnel. The organic phase was washed twice with distilled H_2_O (2x). Finally, the filtrate obtained was concentrated by evaporating the solvent, resulting in a viscous mixture. It was then purified using a column packed with silica gel, and an eluent system of n-hexane: ethyl acetate (15:85 to 40:60 (v: v) was used in a gradient manner.

## Physical characteristics and spectral data for MBH adduct 5-8 (known compounds):

## Compound 5: Methyl 2-(hydroxymethyl) acrylate:

Characteristics: Uncolored concentrated oil, b.p: 97-98°C; IR spectrum, V‾ , cm^-1^: 340 (-O-), 3050 (=CH-)- 2952 (sp3 C-H), 1631 (C=O), and 1718 (C=C).

## Compound 6: Methyl 2-(hydroxy(p-tolyl)methyl)acrylate:

Characteristics: Brown viscous oil, b.p: 109-111°C; IR spectrum, V‾ , cm^-1^: 343 (-OH), 2956 (sp3 C-H), 2853(sp3 C-H), 1724 (C=O), 1632 (C=C), 1258, and 1082.

## Compound 7: Methyl 2-(hydroxy(4-nitrophenyl)methyl)acrylate:

Characteristics: Dark red solid, m.p: 113-115°C; IR spectrum, V‾ , cm^-1^: 312 (-OH), 2952 (sp3 C-H), 2893(sp3 C-H), 2851(sp3 C-H), 1738 (C=O), 1641 (C=C), 1539 (Aromatic C=C), and 1378.

## Compound 8: 2-[(4-Chloro-phenyl)-hydroxy-methyl]-acrylic acid methyl-ester:

Characteristics: Dark yellowish viscous oil, b.p: 111-112°C; IR spectrum, V‾ , cm^-1^: 332 (-OH), 2954 (sp3 C-H), 2833(sp3 C-H), 1717 (sp3 C-H), 1612 (C=O), 1511 (Aromatic C=C), and 1251.

## Procedure and spectral data for the preparation of amide 12-15 from MBH adducts:

Toluene dissolved in one equivalent of MBH adduct substrate was placed in a 50-100 mL round bottom flask and added 1.2 equivalent of the respective amine was added. A catalytic amount of sodium methoxide was added to the reaction mixture, which was then stirred and refluxed. The progress of the reaction was confirmed by TLC analysis. After 15 h, a small, more polar spot was observed on the TLC card along with the reactant spot, and the spot increased after 20 h, but remained constant after 20 h until 24 h. Then, the reaction was subjected to workup, that is, excess solvent was evaporated, and the obtained concentrated material was mixed with a suitable amount of EtOAc and washed twice with distilled water. The organic phase was separated, concentrated, and subjected to chromatographic column purification using EtOAc and hexane (20:80 gradient) as the eluent system. The desired products were obtained after concentrating the desired fraction of the chromatographic column. The pure products were further characterized using spectroscopic techniques.

## Compound 12: 2-(2-(hydroxy(4-nitrophenyl)methyl)acrylamido)acetic acid:

Characteristics: Redish solid, m.p: 121-123°C: IR, V‾ , cm^-1^: 3316 (-OH), 3165 (-NH-), 2950 (sp3 C-H), 2856 (sp3 C-H), 1704 (C=O), 1648, 1519, 1479, and 1346; 1H-NMR, δ, ppm= 11.2 (s, 1H), 8.21 (d, 2H), 8.04 (s, 1H), 7.56 (d, 2H), 5.7 (s, 1H, vinyl), 5.5 (s, 1H, vinyl), 5.12 (s, 1H), 3.61 (s, 2H); HRMS Calculated for C_12_H_13_N_2_O_6_ [M + H]+ 281.080, found 281.102.

## Compound 13: 2-(hydroxy(4-nitrophenyl)methyl)-N-propylacrylamide:

Characteristics: Dark red gummy solid, m.p: 118-120°C: IR (V‾ = cm^-1^): 3270, 2955, 2856, 1712, 1680, 1613, 1558, 1381, and 1311; 1H-NMR, δ, ppm= 8.14 (d, 2H), 7.95 (s, 1H), 7.43 (d, 2H), 5.7 (s, 1H, vinyl), 5.5 (s, 1H, vinyl), 5.12 (s, 1H), 5.27 (s, 1H), 2.95 (t, 2H), 1.60 (quin, 2H), 0.95 (t, 2H); HRMS Calculated for C_13_H_17_N_2_O_4_ 265.110 [M + H]+, found 265.430.

## Compound 14: 2-(hydroxy(p-tolyl)methyl)-N-propylacrylamide:

Characteristics: Brown gummy solid, m.p: 116-118°C; IR (V‾ = cm^-1^): 3371, 2951, 2855, 1708, 1618, 1482, and 1291; 1H-NMR, δ, ppm= 8.14 (d, 2H), 7.95 (s, 1H), 7.43 (d, 2H), 5.7 (s, 1H, vinyl), 5.5 (s, 1H, vinyl), 5.12 (s, 1H, vinyl), 5.27 (s, 1H), 2.95 (t, 2H), 1.60 (quin, 2H), 0.95 (t, 2H); HRMS Calculated for C_14_H_20_NO_2_ [M + H]+ 234.150, found 234.320.

## Compound 15: N-Benzyl-2-[(4-chloro-phenyl)-hydroxy-methyl]-acrylamide:

Characteristics: Brownish solid, m.p: 128-130°C; IR (V‾ = cm^-1^): 3355, 2962, 1711, 1621, 1505, and 1310; 1H-NMR, δ, ppm= 8.15 (s, 1H), 7.19 (d, 2H), 7.10 (d, 2H), 7.08 (d, 2H), 7.15 (m, 3H), 5.61 (s, 1H) 5.42 (s, 1H), 5.10 (bs, 1H), 4.1 (s, 2H), 2.51 (s, 1H); HRMS Calculated for C_17_H_17_ClNO_2_ [M + H]+ 302.110, found 302.126.

## Molecular docking:

The proteins used in the docking study were obtained from the Protein Databank (PDB code 1XPB). The structures of the compounds were sketched using the chemical structure drawing package ChemOffice 8.0. Energy minimization was performed using the MM2 force field. 10,000 iterations were performed with a convergence criterion of 1.000 kcal/atom/ps. AutoDock Vina [14] with global search and internal heuristic algorithms was used to dock the ligands in the binding site of the bacterial enzyme *β-lactamase*. Docking speed is important in the careful study of even a small set of compounds. Default parameters were used in all the docking experiments. During the docking experiment, the enzyme structure was checked for missing atoms. AutoDock Tools was used to assign polar hydrogens, united atom Kollman charges, solvation parameters, and fragmental volumes. The grid for docking calculations was centered to cover the binding site residues. The grid box was calculated by employing compound structures to minimize computation time. A number of points were placed in xyz of a 50 x 50 x 50 Å box to enclose the ligand. The box spacing was 1 Å, and the grid center was designated at dimensions (x, y, z): 16.106, 7.589, and 13.124. AutoDock Vina was executed using protein and ligand information along with the grid box properties in the configuration file. The results of than 1.0 Å in positional root-mean-square deviation (RMSD) were clustered together and represented by the result with the most favourable free energy of binding. The pose with the lowest binding energy or binding affinity was extracted and aligned with the receptor structure for further analysis.

## Pharmacokinetic properties:

The drug-likeness properties of the synthesized compounds were investigated using SwissADME [15] and Molinspiration web-based software.

## Results:

The MBH reaction between aldehydes and methyl acrylate results in a series of MBH adducts, as shown in general scheme 1(see PDF) and in Table 1.

After spectral characterization of the prepared MBH adducts, subsequent amidation (under optimized conditions) of these adducts (6-8) resulted in a series of amides, as shown in general scheme 2(see PDF) and Table 2.

After successful synthesis and characterization of the MBH adducts (5-8) and respective amides (12-15), a molecular docking study was performed. The results obtained are shown in Table 3.

Furthermore, the absorption, distribution, metabolism, and excretion (ADME) properties were also observed for all the synthesized MBH adducts and amides using SwissADME and Molinspiration Software and the results are shown in Table 4.

## Discussion:

## *Preparation and Spectral Analysis of MBH Adducts 5-8*:

Our work begins with the preparation of ester precursors (MBH adducts) to transform them into their respective target molecules, that is, amides. Thus, the previously established procedure reported by us and others [16] was followed. Accordingly, the respective aldehydes were mixed with methylacrylate under basic conditions, that is, by using DABCO as the base and sonicating the reaction at room temperature. Three aldehydes were used separately to obtain the respective MBH adducts 5-8 as shown in Table 1. All the prepared adducts were known compounds. Their structural confirmation was carried out by comparing their IR spectral data with literature data [17]. In addition, TLC analysis suggested their formation. Considering the IR data for adduct 5, a broad signal appeared at 3440 cm^-1^ corresponding to the-OH group, while the signals at 3050 cm^-1^ and 2952 cm^-1^ were assigned to sp2 C-H and sp3 C-H, respectively. The respective C=C and C=O groups gave rise to peaks at approximately 1631 cm^-1^ and 1718 cm^-1^, respectively. The synthesized MBH adduct 6 exhibited IR peaks corresponding to the major functionalities, that is, peaks corresponding to -OH and sp3 C-H, which emerged at 3443 cm^-1^ and 2956 -2853 cm^-1^ respectively. The signals observed at 1724 cm^-1^ and 1632 cm^-1^ were assigned to the respective carbonyl and alkenic functional groups. Compound 7 was obtained as a dark red solid. The major functionalities of this compound were determined by IR spectroscopy. In the IR spectrum, the peaks responsible for sp2 C-H and sp3 C-H were observed at 3512 cm^-1^ and 2893 cm^-1^, 2851 cm^-1^. Other important functional groups such as C=O and C=C bonds gave rise to peaks at 1738 cm^-1^ and 1641 cm^-1^, respectively. The signal at 3512 cm^-1^ suggested the presence of an-OH group. Compound 8 was obtained as dark yellowish viscous oil that showed 111-112°C as with boiling point. In the IR spectrum of compound 8, we observed all relevant peaks where the major functionalities, such as -OH, C=O, and C=C, showed prominent peaks at 3432 cm^-1^, 1717 cm^-1^, 1612 cm^-1^, and 1511 cm^-1^.

## *Synthesis of amides from MBH adducts*:

The prepared MBH adducts were subjected to an amidation reaction, targeting the conversion of their ester functionalities into their respective amides. For this purpose, we initiated amidation with one of the commonly used literature methods, that is, direct amidation of esters with amines catalyzed by sodium methoxide [18]. Thus, in the first attempt, amidation over MBH adduct 5 (an aliphatic adduct) was performed. Adduct 5 was dissolved in toluene using 5 mol. % sodium methoxide. It was separately treated with different amines for different time intervals and at different temperatures to check whether the reaction worked and, if yes, to what extent. In this context, Table 5 shows the various conditions employed during the amidation of aliphatic MBH adduct 5.

**Figure 3:**
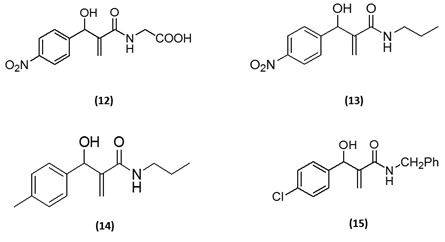
Structural representation of the synthesized MBH adduct's derived amides 12-15.

At the initial observation of the TLC analysis, an expected and comparatively slight polar spot did not appear on the TLC card. Additionally, the IR spectra observed for the expected products given in the above table remain the same as those of the reactant, which suggests that aliphatic MBH adduct 5 did not transform into their expected products 9-11.This method was then applied to the transformation of aromatic MBH adducts into their amide derivatives. In this context, 1 eq. of each reactant adduct was primarily dissolved in toluene and then treated with the respective amines, and the reaction mixture was stirred and refluxed (Table 2.). Considering the formation of compound 12, the initial TLC analysis revealed the formation of the expected products as a slightly polar spot compared to the reactants on the TLC card in a system of ethyl acetate and n-hexane in a ratio of 18:82. The obtained products were further confirmed by spectral analysis. The yield obtained under the given reaction conditions was 20%, while the yield increased when NaOMe was used along with the other reaction conditions as mentioned in Table 2. The synthesized aromatic aldehyde based amide products 12-15 are also presented in Figure 3.

**Figure 4:**
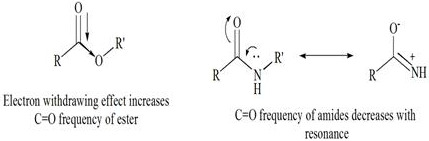
IR frequencies variation due to resonance in esters and amides

To characterize compound 12, IR spectra were recorded, where the peak responsible for its -OH group appeared at 3316 cm^-1^ and a newly developed peak compared to the reactant spectra appeared at 3165 cm^-1^ that was attributed to-NH group of the product amide. The absorption peak due to-C-H stretching was observed in the range of 2950-2856 cm^-1^. An interesting peak observed for the carbonyl of amide 12 (product) at 1704 cm^-1^ was a clear indication of product formation. This is because the peak observed for the same functionality (C=O) in the reactant appeared at 1738 cm^-1^, which shows a shift of the frequency from higher (for the substrate) to lower (for the product) [19]. This rationality of frequency is strongly supported by the literature (Figure 4).

Literature report revealed that amide bonds weaken the strength of carbonyl bonds through resonance, and vice versa. Accordingly, in the case of amide vs. ester carbonyl, the amide yields a relatively lower IR spectral value. Additional peaks observed at 1648 cm^-1^, 1519 cm^-1^ and 1479 cm^-1^ suggested the presence of C=C bonds in the allylic and aromatic systems, respectively. The 1H-NMR spectrum exhibited all the relevant peaks such as a singlet at 11.2 ppm was assigned to acidic proton, the doublets appeared at 8.21 and 7.56 indicated aromatic protons while the peak at 8.04 ppm was assigned to NH proton. Similarly, the vinylic protons appeared at 5.7 and 5.5 ppm while a singlet observed at 5.12 ppm was linked to benzylic proton. In addition, a singlet at 3.61 appeared which is attributed to the methylene proton at the alpha position of the carboxylic acid functionality. The mass spectral results for product 12 revealed the presence of a molecular ion peak at 281.10, which suggested its molecular formula as C_12_H_12_N_2_O_6_.

Compound 13 was obtained from the respective MBH adduct as a dark-red gummy solid. In this case, the amine used was an aliphatic primary amine carrying an alkyl-donating group, unlike the acidic group of glycine (Table 2). TLC analysis revealed the conversion of the starting material after 17 h, and the spot (slightly polar spot for the desired product) became more visible after 20 h. The structural and molecular formula of amide 13 was confirmed by IR, NMR, and mass spectral analyses. Considering the IR spectra of compound 13, a distorted broad peak at 3270 cm^-1^ indicates the -OH and -NH groups of the amide product. Other peaks observed at 1680 cm^-1^, 1558 cm^-1^, and 1381 cm^-1^ corresponded to allylic and aromatic unsaturation, while an important peak (shifted to the lower frequency, similar to that observed previously for reactant 7) was observed at 1712 cm^-1^, which was assigned to the respective carbonyl of the product amide 13. In 1H-NMR spectrum for the compound 13 we observed aromatic protons at 8.14 and 7.43 as doublets, a singlet for NH at 7.95ppm and two singlets at 5.7, 5.5 ppm was assigned to vinylic protons. The bezylic proton appeared at 5.12 ppm while a triplet, quintet and a triplet at 2.95, 1.60, 0.95 ppm. Considering the mass spectra, the respective M+ at 265.430 suggested a molecular formula of C_13_H_17_N_2_O_4_. Compound 15 was obtained as a brown solid using the same procedure as that used for the formation of compound 12-14. The melting point of this compound was determined as 128-130°C. Spectral studies have suggested its molecular and structural formulas. According to the molecular ion peak observed at m/z = 302.126, the molecular formula of compound 15 was suggested to be C_17_H_16_ClNO_2_. The structure of this compound, shown in Table 2, was suggested by its IR and NMR spectra. In the IR spectrum, the peaks appearing at 3355 cm^-1^, 1711 cm^-1^, 1621 cm^-1^, and 1505 cm^-1^ indicate the presence of -OH, C=O, and C=C, respectively. In the NMR spectrum of compound 15, we observed the NH proton at 8.15 ppm, aromatic protons in a rage of 7.19-7.08 ppm while the venilic and benzylic protons were appeared at shift ranging of 5.61 - 4.1 ppm.

## Molecular Docking:

**Figure 5:**
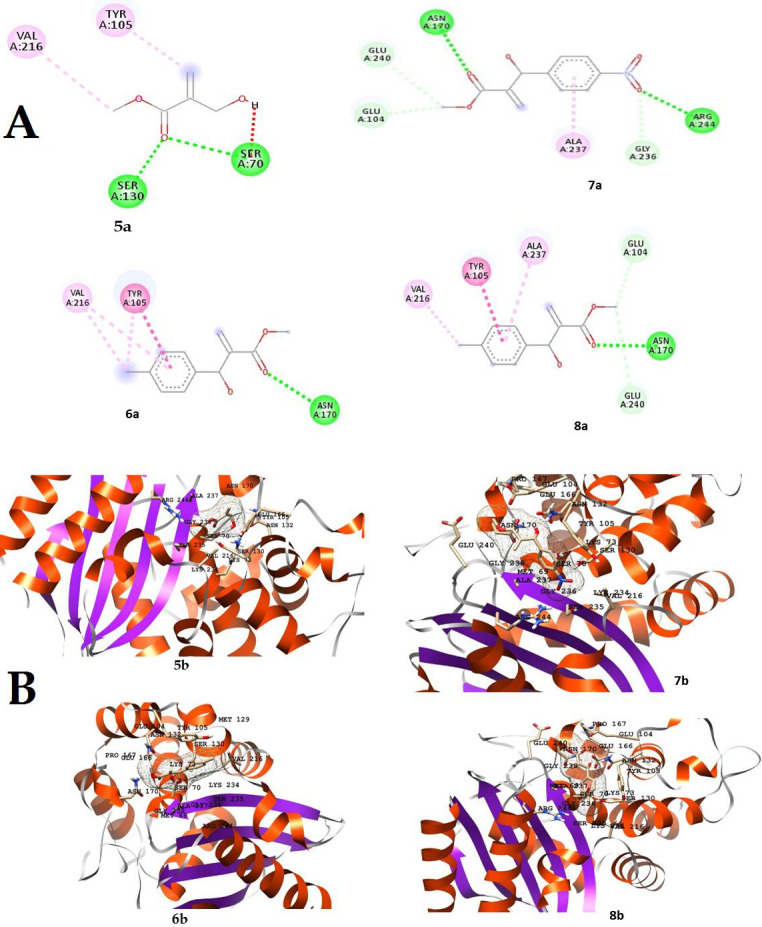
(A) 2D representation of the docked MBH adducts (6-8), indicating interaction with the active sites of β- lactamase; Figure (B) 3D representation of the docked MBH adducts (6-8), indicating interaction with the active sites of β- lactamase

**Figure 6:**
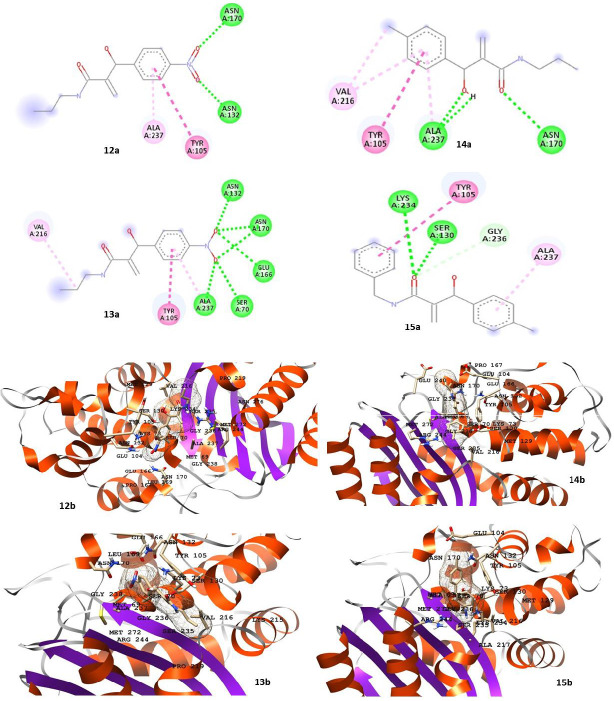
(A) 2D representation of the docked MBH adducts (12-15), indicating interaction with the active sites of β- lactamase; (B) 3D representation of the docked amides (12-15), indicating interaction with the active sites of β- lactamase. A B

Molecular docking studies were performed on the newly synthesized compounds with the bacterial target enzyme *β-lactamase* to provide a mechanistic explanation for the antimicrobial activity of the binding complexes. This *in silico* study will provide further insight into the binding energies and intermolecular interactions in the active pocket of the enzyme. Chloramphenicol and paracetamol were used as standard compounds for the *in silico* screening of the newly synthesized compounds. The docking study was performed using AutoDock Tools and AutoDock Vina. The molecular docking technique was performed in rigid docking mode, and nine conformations were generated for each docked molecule [20]. The results obtained from the docking studies of the synthesized compounds are presented in Table 3). The results indicate that all synthesized compounds fit well inside the active site of the enzyme (Figure 5 & Figure 6). The docked compounds were ranked according to their binding energies to the active site of the target protein. Compound (15) exhibited a good docking score (-8.6 kcal/mol) as well as good intermolecular interaction networks such as H-bond, π-π, and π-alkyl stacking with the amino acid residues Ser70, Tyr105, Ser130, Asn132, Val216, Ser235, Ala237, Gly236, and Arg244. It was also inferred from the results that compound 15 demonstrated the best docking score (-8.6 kcal/mol) among all the synthesized compounds, even higher than the standard drugs (as mentioned in Table 4) against the target enzyme. The 2D and 3D representation of the docked MBH adducts are shown in Figure 5A and Figure 5B, respectively, while their respective docked products (amides 12-15) are presented in 2D and 3D forms in Figure 6A and Figure 6B, respectively.

## Prediction of absorption, distribution, metabolism, excretion (ADME) properties:

The ADME test was performed using SwissADME and Molinspiration to determine the pharmacokinetic properties of all synthesized compounds. The results indicated that all synthesized compounds showed drug-likeness properties, as suggested by Lipinskin's rule of five. The ADME properties are listed in Table 4.

## Conclusion:

Studies on the direct amidation of ester functionalities containing MBH adducts have been carried out, and as a result, one aliphatic and three aromatic adducts 5-8. Among the three aliphatic adducts, 5 was subjected to amidation to achieve Amides 9-11 but the reactions were unsuccessful. Then, aromatic adducts 6-8 were successfully transformed into Amide 12-15 through an amidation reaction using sodium methoxide as a base and toluene as a solvent. This indicates the smooth reactivity of aromatic adducts towards amidation. It was also inferred that the adduct bearing an electron-withdrawing group transformed into its respective amide with a better yield than that bearing the donating group. Additionally, molecular modelling studies were performed on the synthesized MBH adduct precursors and their derived amides to examine their binding affinity for *β-lactamase*. The results indicated that all the synthesized compounds showed significant binding affinity with the selected enzyme (*β-lactamase*), where MBH adduct 7 and its derived amide 15 showed the highest docking scores, thus proving to be good inhibiting agents for *β-lactamase* and thus good antibacterial agents. In the future, the synthesis of further examples and their complete bioassay (*in silico*, *in vitro*, and *in vivo*) profiles are planned to achieve more potent antibacterial agents. Such studies can contribute significantly to the discovery of new drugs or drug precursors.

## Funding:

The authors declare that no funds, grants, or other support were received during the preparation of this manuscript.

## Author's contributions:

Conceptualization & supervision: Hamid Ullah and Sadia Majeed; experimental studies and methodology: Sadia Majeed, Asma Abro, and Mehboob Ahmed; validation: Taj Ur Rahman and Abdul Majedd Khan; writing-original draft preparation: Muhammad Asif, Asma Yousafzai, and Riffat Ullah; writing-review and editing: Mahmood Rasool, and Peter Natesan Pushparaj. All the authors have read and agreed to the published version of the manuscript.

## Data availability:

The data supporting the results are described in the figures and tables given in the manuscript, while further data in support of the results will be provided by the authors upon responsible request.

## Figures and Tables

**Table 1:** Synthesized MBH adducts

**Aldehyde (Reactant)**	**MBH adduct (Product number)**	Time (hrs)/% Yield*
Formaldehyde	2-Hydroxymethyl-acrylic acid methyl ester (5)	120/ 74
4-Methyl benzaldehyde	2-(Hydroxy-*p*-tolyl-methyl)-acrylic acid methyl ester (6)	144/73
4-Nitrobenzaldehyde	2-[Hydroxy-(4-nitro-phenyl)-methyl]-acrylic acid methyl ester (7)	48/94
4-Chlorobezaldehyde	2-[(4-Chloro-phenyl)-hydroxy-methyl]-acrylic acid methyl ester (8)	52/85
* The yield shown is for the pure product

**Table 2:** Synthesis of amide 12-15 from adducts 6-8

**Reactant Amine**	**Catalyst**	**Product Amides (12-15)**	**Time/% Yield**
Glycine	Nil	{2-[Hydroxy-(4-nitro-phenyl)-methyl]-acryloylamino}-acetic acid(12)	24 hrs/20
	NaOMe		24 hrs/68
n-Propylamine	NaOMe	2-[Hydroxy-(4-nitro-phenyl)-methyl]-N-propyl-acrylamide(13)	24 hrs/74
n-Propylamine	NaOMe	2-(Hydroxy-p-tolyl-methyl)-N-propyl-acrylamide(14)	24 hrs/70
Benzyl	NaOMe	N-Benzyl-2-[(4-chloro-phenyl)-hydroxy-methyl]-acrylamide(15)	20 hrs/72

**Table 3:** Interaction and binding energies of the synthesized compounds in the active site of the target protein (*β- beta-lactamase*)

**S. No**	**Compound name (No)**	**Docked complex interaction**	**Distance (Å)**	**Binding Affinity (kcal/mol)**
1	5	Hydrogen bonding		-4.9
		Ser130:HG - O	1.96	
		Ser70:HG - O	2.28	
		Ser20:HB2 - O	3	
		Pi-Alkyl		
		Tyr105	3.9	
		Alkyl		
		Val216	4.98	
		Unfavorable donor-donor interaction		
		Ser70:HG - H	1.41	
2	6	H-bond		-7.5
		ASN170:HD	2.77	
		Pi-pi stacked		
		Val216	5.29	
		Tyr105	3.95	
		Pi-alkyl		
		Val216	4.65	
		Tyr105	4.67	
3	7	H-Bonding		-7.9
		Gly236:HA1 - O	2.96	
		ARG244:HH21 - O	2.83	
		Asn170:HD22 - O	2.42	
		Glu104:OE2	3.48	
		Glu240:OE2	3.6	
		Arg244:HH22 - O	2.64	
		Pi-Alkyl		
		Ala237	4.54	
4	8	H-bond		-6.9
		Asn170:HD22 - O	2.64	
		Glu240:OE2	3.66	
		Glu104:OE2	3.57	
		Alkyl		
		Val216	4.48	
		Pi-pi stacked		
		Tyr105	5.03	
		Pi-alkyl		
		Ala237	4.66	
5	12	Hydrogen bond		-7.5
		Asn170:HD21 - O	2.43	
		Asn132:HD21 - O	2.06	
		Pi-Alkyl		
		Ala237	4.69	
		Pi-pi stacked		
		Tyr105	5.1	
6	13	Hydrogen bond		-7.5
		Asn170:OD1 - O	3.05	
		Ser70:HN - O	2.37	
		Glu166:OE1 - O	3.38	
		Ala237:O - O	2.89	
		Asn132:HD21 - O	2.83	
		Asn132:OD1 - O	3.12	
		Pi-Alkyl		
		Ala237	4.8	
		Pi-pi stacked		
		Tyr105	4.89	
		Alkyl		
		Val216	4.37	
7	14	Hydrogen bond		-7
		Asn170:HD21 - O	3.02	
		Ala237:O - H	2.76	
		Ala237:HN - O	2.11	
		Pi-Alkyl		
		Ala237	4.88	
		Val216	5.15	
		Val216	3.84	
8	15	Hydrogen bond		-8.6
		Gly236:HA1 - O	2.98	
		Lys234:HZ1 - O	3.96	
		Pi-pi T-shaped		
		Tyr105	5.01	
		Pi-Alkyl		
		Ala237	5.18	
		**Binding affinities of the standard drugs**		
**S. No.**	**Compound Name**		**Binding affinity (kcal/mol)**	
1	Paracetamol		-5.3	
2	Chloramphenicol		-6.7	
3	Benzipram		-7.1	

**Table 4:** ADME properties of the synthesize compounds

**Compound**	**5**	**6**	**7**	**8**	**12**	**13**	**14**	**15**
Molecular weight g/mol	116.12	192.21	238.22	226.66	265.29	265.29	233.31	307.82
Blood-brain Barrier permeant	No	Yes	No	Yes	No	No	Yes	Yes
LogP	0.21	1.65	-0.42	2.19	-0.46	-0.46	2.27	3.42
TPSA A^2^	46.53	46.53	96.19	46.53	98.99	98.99	49.33	49.33
HBA	3	3	5	3	4	4	2	2
HBD	1	1	2	1	3	3	2	2
N rotatable	3	4	5	4	7	7	6	6
Lipinski Violations	0	0	0	0	0	0	0	0
Volume A^3^	109.72	181.15	190.84	194.69	223.45	223.45	234.73	288.34
Bioavailability score	0.55	0.55	0.55	0.55	0.55	0.55	0.55	0.55
GI Absorption	High	High	High	High	High	High	High	High

**Table 5:** Conversion of MBH adduct 5 into amides by treatment with different amines

	**Reactant Amine**	**Catalyst/Solvent/Time/Temp.**	**Expected Amide (9-11)**	**(% conversion)**
1	Glycine	5 mol% NaOMe/ Toluene/10hrs/ 50°C	(2-Hydroxymethyl-acryloylamino)-acetic acid(9)	Nil
		5 mol% NaOMe/ Toluene/15hrs/ 80°C		
		5 mol% NaOMe/ Toluene/24 hrs/ reflux		
2	n-Propylamine	5 mol% NaOMe/ Toluene/10hrs/ 50°C	2-Hydroxymethyl-N-propyl-acrylamide(10)	Nil
		5 mol% NaOMe/ Toluene/15hrs/ 80°C		
		5 mol% NaOMe/ Toluene/24 hrs/ reflux		
3	Benzylamine	5 mol% NaOMe/ Toluene/10hrs/50°C	N-Benzyl-2-hydroxymethyl-acrylamide(11)	Nil
		5 mol% NaOMe/ Toluene/15hrs/ 80°C		
		5 mol% NaOMe/ Toluene/24 hrs/ reflux		
